# How does membership in local savings groups influence the determinants of national health insurance demand? A cross-sectional study in Kisumu, Kenya

**DOI:** 10.1186/s12939-018-0889-7

**Published:** 2018-11-20

**Authors:** Tessa Oraro, Kaspar Wyss

**Affiliations:** 1 Swiss Tropical and Public Health Institute, Basel, Switzerland; 2 University of Basel, Basel, Switzerland; 3 Department of Epidemiology and Public Health, Health Systems Support Unit, Swiss Tropical and Public Health Institute, P.O. Box 4002, Basel, Switzerland

**Keywords:** ROSCAs, Health insurance, Social capital, Kenya

## Abstract

**Background:**

Rotating savings and credit associations (ROSCAs) are highly active in many sub-Saharan African countries, serving as an important gateway for coping with financial risk. In light of the Kenya’s National Hospital Insurance Fund’s (NHIF’s) strategy of targeting ROSCAs for membership enrolment, this study sought to estimate how ROSCA membership influences the determinants of voluntary health insurance enrolment.

**Methods:**

A cross-sectional survey of 444 households was carried out in Kisumu City between July and August 2016. A structured questionnaire was administered on health insurance membership, household attributes, headship characteristics and health-seeking behaviour. We assessed the influence of ROSCA membership on the associations between NHIF enrolment and the explanatory variables using univariate logistic regression.

**Results:**

The study found that education was associated with NHIF demand regardless of ROSCA membership. Both ROSCA and non-ROSCA households with high socioeconomic status showed stronger health insurance demand compared with poorer households; there was, however, no evidence that the strength of this association was influenced by ROSCA status (*p*-value = 0.47). Participants who were self-employed were significantly less likely to enrol into the NHIF if they did not belong to a ROSCA (interaction test *p*-value = 0.03). NHIF enrolment was found to be lower among female-headed households. There was a borderline effect of ROSCA membership on this association, with a lower odds ratio amongst non-ROSCA members (*p*-value = 0.09): the low treatment numbers amongst the insured infers that ROSCA membership may play a role on the association between gender and NHIF demand.

**Conclusions:**

Our findings suggest that ROSCA membership may play a role in increasing health insurance demand amongst some traditionally under-represented groups such as women and the self-employed. However, the strategy of targeting ROSCAs to increase national health insurance enrolment may yield exiguous results, given that ROSCA membership is itself influenced by several non-observable factors – such as time-availability and self-selection. It is therefore important to anchor outreach to ROSCAs within a broader, multi-pronged approach that targets households within their social, economic and political realities.

## Introduction

Informal economic networks based on geographical, sociocultural or occupational proximity are highly active in many sub-Saharan African countries, with an estimated 24% of the population participating in local savings clubs in 2014 [9]. In Kenya, where 24.6% of the population is excluded from formal savings and loans institutions, local rotating savings and credit associations (ROSCAs) have emerged as important gateways for coping with financial instability [7]. ROSCAs are typically created to meet an unmet social and economic need amongst vulnerable groups such as the working poor and women [2, 9], with participants pooling their financial resources to create communal savings funds as a form of financial risk protection [42]. In this sense, ROSCAs serve as social networks through which individuals obtain financial security through collective resource mobilisation. Due to their informal nature, ROSCAs’ success is contingent upon the trust, reciprocity and collective strength shared amongst group members – fundamental components of a phenomenon known as social capital [12, 42]. It is this reliance on social capital that has driven the use of similar welfare associations as launchpads for the national health insurance schemes in Germany and Japan [6, 36].

Against the backdrop of increasing ROSCA membership across sub-Saharan Africa, financial risk protection in health care has remained low. Several governments in the region such as those in Ghana and Kenya have sought to promote national health insurance schemes as a means of minimising the high out-of-pocket costs associated with ill health [38, 39]. In Kenya – where up to 28% of slum-dwelling households face catastrophic health expenditure [5] – the national government has established the National Hospital Insurance Fund (NHIF) as a means of providing financial risk protection to its citizens. The NHIF operates like most national health insurance schemes in the region, with the government and the formal sector providing the bulk of financial contributions to the scheme [40]. However, given the high levels of informal employment in the Kenyan economy, the depth of insurance coverage has remained low, with only 13.5% of the population receiving NHIF coverage [20]. A large proportion of Kenya’s informally employed households – who constitute 85% of the working population – are thus left susceptible to financial shocks in the event of ill health [20, 41]. This problem echoes findings across health insurance schemes in several sub-Saharan countries, where low health insurance demand has been attributed to high levels of labour informality.

As part of efforts to better understand the reasons behind the low uptake of voluntary health insurance, several research studies have identified a link between social solidarity and health insurance demand across various sub-Saharan African settings [1, 14]. This growing body of evidence has led Kenyan policymakers to target ROSCAs as a means of increasing NHIF demand amongst the informal sector, in line with their role as vehicles for social solidarity [33]. Consequently, we seek to understand how ROSCA membership influences the drivers of voluntary health insurance in Kisumu, Kenya. This paper will be structured as follows: We will first provide a review of the research on the intersection between local group membership, social capital, and voluntary health insurance membership. We will subsequently report our methodology and results, and conclude with an analysis of our findings.

In order to better understand the mechanisms through which ROSCAs may influence health insurance enrolment, it is first necessary to define the social capital underpinning the success of ROSCAs. Social capital as a concept focuses on the utility of relationships and resources in facilitating collaboration towards a common goal [4, 8, 34]. While different interpretations of its nature and effect exist, it is generally agreed that social capital influences the depth of individuals’ engagement with local and national structures by facilitating trust and reciprocity within communities. ROSCAs – as local informal groupings – seek to empower economic cooperation in the face of complex resource and capacity constraints. In doing so, they tap into two key dimensions of social capital: intra- and inter-community collaborations. When constituting ROSCAs in low-resource settings, members often recruit those in whom they have inherent trust – ostensibly those with similar socioeconomic, professional or ethno-cultural characteristics. This structure mimics traditional African obligations to familial and tribal alliances [3], and derives its power from harnessing existing links within homogenous groups: a concept known as bonding social capital [30]. Concurrently, in order to make ROSCAs sustainable, members seek to increase the associations’ capital base and minimise the risk of default [2]. They thus expand their radius of trust beyond their typical personal, sociocultural and organisational bases - a phenomenon known as bridging social capital [30]. In this vein, ROSCAs foment economic, social, and cultural links within local communities [35].

In spite of the pervasiveness of ROSCAs and their linkages to national health insurance enrolment in several sub-Saharan African settings [24, 27], there remains a paucity of research on the influence of ROSCAs on national health insurance demand. We can, however, draw lessons from studies carried out on the influence of social capital as a general concept on voluntary health insurance enrolment. According to existing research, active members of local mutual assistance groups such as ROSCAs are more likely to be part of voluntary health insurance schemes due to a shared ethos of social cooperation [11, 15, 31]. These findings highlight the importance of common norms, values and objectives – characteristic features of social capital – in influencing households’ decision to enrol into voluntary health insurance. Given this, our paper will seek to answer the following research question: How does ROSCA membership influence the determinants of voluntary health insurance enrolment? We will further seek to understand how existing knowledge on social capital can help explain the influence of ROSCAs on the determinants of health insurance demand.

In order to answer these questions, we can apply several hypotheses to our study based on the findings of existing research. Given the link identified between local informal group membership and social capital, we anticipate that non-ROSCA members may have weaker bonding and bridging social capital compared with their ROSCA member counterparts. As a result of this, we expect wealth to manifest as an overarching determinant of NHIF demand amongst non-ROSCA members in line with existing national health financing studies [10, 17, 18, 24, 26, 27]. Additionally, we hypothesise that sociocultural sensitivities such as household composition; gender of household head; and marital status will influence households’ likelihood of enrolling into national health insurance [10, 17, 18, 24–27]. We however postulate that these inequities may be reduced by ROSCA membership due to their high social capital stock.

## Methodology

### Sampling methodology

A cross-sectional household survey was carried out in Kisumu City between July and August 2016 to identify the extent to which NHIF enrolment is influenced by ROSCA membership; household composition and attributes; as well as household head characteristics such as occupation, age, gender and perceived health status of household members.

Our study aimed to estimate the proportion of the informally employed population with voluntary NHIF health insurance, and to assess the association between explanatory variables and this proportion. In order to achieve these aims, we calculated the sample size based on the ability to estimate the proportion insured with a certain precision - in this case, with an expected NHIF population coverage of 13.8% [29]. Using the Hayes and Bennett equation and taking into account clustering in six sub-locations across Kisumu, required sample size was calculated to be 440 households with the precision of a 95% confidence interval with a width of 10 to 18% [16]. The number of households interviewed in each sub-location was proportional to its demographic size, which was obtained from the 2009 Kenya Population and Housing Census [22].

Multistage cluster sampling was used to obtain the study sample, with enumeration maps of the six sub-locations used to randomly identify existing informal settlements in Kisumu [19]. In the second stage, we selected existing water points within informal settlements, and randomly selected starting points located near the water points. This strategy was driven by the use of watering points as navigation tools by the local communities. We subsequently carried out systematic sampling, with every nth household interviewed. Approximately 98% of the targeted households could be interviewed.

### Data collection

The survey was administered as a structured questionnaire using Open Data Kit (ODK) software on handheld tablets to answer questions on household composition; household assets; household expenditure and consumption; and health-seeking behaviour. Research assistants who were fluent in English, Kiswahili and the local dialect, Luo, and who had knowledge of the local geographical and sociocultural context were hired. Training was provided to familiarise research assistants with the questionnaire and data collection using handheld tablets.

Household heads were targeted as the primary respondents for this study. In cases where the household head was not available, their spouse was interviewed.

In order to identify the informally employed in our study area, we used a deductive approach of identifying households where the household head did not make mandatory monthly payments into the National Social Security Fund (NSSF). This approach was taken, as there is no clearly defined approach for identifying the informal sector in Kenya. However, the formal sector is defined distinctly within Kenya’s legal framework, with sector members legally obligated to make monthly pension payments to the NSSF [32].

Insured households were defined as those where members were voluntarily enrolled into the NHIF in the year preceding the study.

### Statistical analysis

The data were analysed using STATA version 14.1 for Windows (STATA Corporation, College Station, Texas). The main outcome variable was voluntary enrolment into the NHIF in the previous year. The explanatory variables were divided into five components: household composition and attributes; household head factors; perceived household health status; and proxies for exposure to financial risk pooling.

In order to measure wealth, we used the asset index as described within the Kenya Demographic and Health Survey (DHS) to calculate households’ asset-based wealth [21]. We collected data on consumer items, dwelling characteristics such as housing materials, water source, sleeping arrangements, and other characteristics linked to wealth status. We subsequently computed the asset index. Households were then classified into one of five socioeconomic groups based on quintiles (Q): Q1 or the poorest 20%; Q2; Q3; Q4; and Q5 or the wealthiest 20% in the sample. These groups were subsequently clustered into three groups: Q1 and Q2; Q3 and Q4; and Q5 respectively, to account for the minimal differences amongst the proximal quintiles. Literacy within the study context was defined as those above the age of 15 years who could read and write [37]. The mean perceived health status of each household was calculated as the average self-reported health status value for all household members. The final value of the mean perceived health status was assigned a value of between 1 and 4, with 1 being “very good” health status; 2 being “good” health status; 3 being “poor” health status; and 4 being “very poor” health status.

We used multilevel mixed-effects logistic regression models to estimate the association between health insurance enrolment and the explanatory variables. We took the clustering of households at the village level into account using random effects for village. In light of the low numbers of insured households within our study population, it was not possible to include multiple covariates simultaneously in the model. Therefore, univariate models were run to estimate the association of each variable and health insurance enrolment for the ROSCA and non-ROSCA households respectively. We ran the regression models for (i) ROSCA and non-ROSCA separately to easily gain estimates of the univariate odds ratios and 95% confidence intervals; and (ii) on the full dataset with ROSCA and non-ROSCA households in order to use interaction terms to directly test whether the associations between each covariate and NHIF enrolment were affected by ROSCA membership. Each model included the covariate, ROSCA membership, and the interaction term as explanatory variables.

## Results

### Study population characteristics

A total of 444 households were enumerated in the survey, with ROSCA member households accounting for 63% of the study population. Approximately 29% of ROSCA households and 23% of non-ROSCA households were voluntarily enrolled into the NHIF. While ROSCA members are actively targeted by the NHIF for enrolment, our study did not find a statistically significant association between ROSCA membership and health insurance enrolment (OR: 1.33 [0.85–2.09]). Table 1 reports the differences in each test variable according to insurance status and household ROSCA membership status.

**Table 1:** Descriptive statistics by gender and insurance status

	ROSCA member			Non-ROSCA member		
	Insured (*n* = 38)	Uninsured (*n* = 125)	Combined (*n* = 163)	Insured (*n* = 81)	Uninsured (*n* = 200)	Combined (*n* = 281)
Household characteristics						
Median household size^b^	4 (3,5)	4 (3,5)	4 (3,5)	5 (3,6)	4 (3,5)	4 (3,5)
Households with children <=5 years	48%	59%	56%	61%	58%	58%
Households with children <=15 years	75%	76%	76%	84%	78%	79%
Households with elderly > = 60 years	11%	6%	7%	8%	10%	10%
Average socioeconomic status (SES) scorea^a^	3.47 (0.28)	2.95 (0.27)	3.19 (0.27)	3.16 (0.21)	2.69 (0.26)	2.80 (0.25)
% of poor households (Q1-Q2)	26%	42%	37%	29%	50%	45%
% of rich households (Q5)	32%	18%	22%	18%	15%	16%
Annual average household expenditure (KShs)^a^	365,060 (154435)	361,195 (734010)	362,309 (624283)	373,513 (571020)	230,524 (208211)	263,859 (333668)
Household head characteristics						
Median age^b^	39 (29,45)	35 (28,40)	36 (27,40)	34 (27,42)	35 (26,39)	35 (27,40)
Female household heads	21%	29%	26%	13%	34%	29%
Secondary-level education and above	63%	49%	53%	71%	43%	50%
Married household heads	83%	72%	75%	87%	66%	71%
Paid employee	38%	38%	38%	61%	30%	37%
Small-scale business owner	56%	57%	57%	32%	62%	55%
No fixed employment	6%	5%	5%	8%	7%	7%
More than one earner	52%	32%	37%	39%	19%	24%
Profits as remuneration	27%	24%	25%	18%	18%	18%
% living below poverty line^c^	14%	20%	19%	39%	38%	38%
Household health status						
Mean health status^a^	2.04 (0.59)	1.96 (0.52)	1.98 (0.54)	2.03 (0.51)	2.04 (0.56)	2.04 (0.55)
Presence of chronic disease	4%	6%	5%	3%	7%	6%
Use of curative services						
Outpatient service use in past 30 days	48%	49%	48%	45%	37%	39%
Inpatient service use in past year	36%	25%	28%	16%	22%	21%
Annual per capita health expenditure (KShs)^a^	1729 (18607)	739 (3542)	1025 (10516)	569 (2946)	818 (6442)	760 (5821)

^a^Mean (±Standard Deviation)

^b^Median (Inter-Quartile Range)

^c^Poverty line defined as households living on less than $1.90 a day

1USD = 102.45 KShs as of January 2018

Most household heads in our study population reported themselves as economically active, with 94% of respondents carrying out casual income-generating activities in lieu of formal employment. The most common economic activities amongst our study population were small-scale business ventures, with 56% of the population engaging in activities such as carpentry, masonry, and hairdressing. This notwithstanding, our findings revealed that 26% of our study population lived below the poverty line, which is defined as a household income of less than $1.90 a day [41].

The household characteristics and health status for the sample population were broadly similar regardless of ROSCA membership status. Overall, the median household size was 4, with 77% of all households having children below the age of 15 years. The number of households with elderly members was low across the study population, with only 8% of households having members above the age of 65 years. The study population also showed similarities in the gender and marital status of household heads, irrespective of ROSCA membership status. Further, the study found similarities in the mean household health status, with 5% of households reporting the presence of chronic disease.

The study found important distinctions in the economic characteristics of ROSCA and non-ROSCA households. ROSCA members were more likely to have more than one earner in the household and higher monthly incomes compared to non-members.

### Factors associated with voluntary health insurance enrolment amongst ROSCA and non-ROSCA member households

Household-level regression results are presented in Table 2 for ROSCA and non-ROSCA member households.

**Table 2:** Univariate logistic estimates for probability of purchasing NHIF health insurance at household level

	ROSCA members (*n* = 163)		Non-ROSCA members (*n* = 281)		Interaction* (*n* = 444)
Variable description	Odds ratio	CI	Odds ratio	CI	*p*-value
Household characteristics					
Presence of children < 15 years	0.92	0.49–1.72	1.54	0.59–4.05	0.50
Presence of elderly > 60 years	1.93	0.76–4.90	0.74	0.20–2.74	0.15
Socioeconomic status (Reference group: First and second quintiles)					
Middle wealth	1.71	0.91–3.23	2.56	1.12–5.88	0.47
Wealthiest quintile	2.81	1.38–5.73	2.08	0.71–6.10	
Household head characteristics					
Sex of household head (Reference group: Male)					
Female	0.65	0.35–1.23	0.29	0.11–0.79	0.09
Age (Reference group: ≤24 years)					
25–29 years	0.71	0.27–1.90	1.29	0.35–4.72	0.16
30–45 years	0.92	0.38–2.22	2.16	0.65–7.17	
46–59 years	1.92	0.68–5.42	1.79	0.41–7.86	
60+ years	1.92	0.59–6.24	0.42	0.04–4.18	
Education (Reference group: Primary level or less)					
Secondary education or higher	1.80	1.05–3.11	3.23	1.47–7.08	0.84
Employment type (Reference group: Paid employees)					
Self-employed	0.95	0.55–1.66	0.25	0.11–0.57	0.03
No fixed employment	1.15	0.35–3.75	0.55	0.14–2.25	
Remuneration (Reference group: Fixed salary)					
Daily/hourly pay	0.70	0.29–1.71	0.20	0.07–0.53	0.19
Task-based payment	0.52	0.23–1.14	0.15	0.05–0.44	
Business profits	0.76	0.33–1.76	0.32	0.11–0.95	
Marital status (Reference group: Unmarried)					
Married	1.87	0.96–3.64	3.46	1.26–9.51	0.57
Presence of chronic illness in household	0.65	0.17–2.44	0.35	0.43–2.84	0.46

*The *p*-values assess whether the association between each variable and NHIF membership is significantly different for the ROSCA and non-ROSCA households

The odds in the reference group are multiplied by the odds ratio for each category

Our study found that education and asset-based SES were associated with NHIF demand, regardless of ROSCA membership status. Household heads in the study population were more likely to enrol into the national health insurance scheme if they were educated to at least secondary school level (OR = 1.80 (CI:1.05–3.11) for ROSCA members, and OR = 3.23 (CI:1.47–7.08) for non-ROSCA members (interaction test = 0.84). Our findings also suggest that SES was associated with health insurance demand, although there was no evidence that the strength of the association was influenced by ROSCA membership status (*p*-value = 0.47).

Our analysis showed that the relationship between NHIF enrolment and some socio-demographic and economic variables could be influenced by a household’s ROSCA status. The study found that household heads that were self-employed or had no fixed employment were significantly less likely to enrol into the NHIF if they did not belong to a ROSCA (*p*-value = 0.03).

Our findings also raise the possibility of a stronger relationship between gender and national health insurance demand if one does not belong to a ROSCA than if one does (*p*-value = 0.09). According to our analysis, non-ROSCA female household heads were less likely to enrol into the NHIF compared with their male counterparts (OR = 0.29 (CI:0.11–0.79). The odds ratio among the ROSCA member group was 0.65 (CI:0.35–1.23). Given the low treatment numbers amongst the insured, the borderline effect of ROSCA membership on the relationship between gender and health insurance demand infers that it may play an influencing role on the association between the explanatory variable and NHIF enrolment.

In addition to the above associations, our findings suggest that NHIF demand may increase if the household head was married or received a fixed salary. There was, however, no evidence that these associations were influenced by ROSCA membership.

## Discussion

This study investigated the influence of informal financial coping mechanisms on the demand for formal, more secure forms of financial risk protection in health. Before probing the associations between ROSCA membership and health insurance enrolment, it is first necessary to examine the overarching determinants of NHIF demand within the general population sample. According to our findings, the NHIF has been successful in providing inclusive health insurance coverage regardless of household composition or health status. This may be attributed to the NHIF’s benefit structure, which enables a household to enrol all its identified dependants under a fixed premium irrespective of their individual circumstances. While successful in expanding coverage amongst the above-mentioned groups in our study area, we found that the NHIF still faces challenges in increasing health insurance demand amongst key vulnerable population groups, including those with low levels of education and limited asset-based wealth. These findings reflect similar national health financing studies across sub-Saharan Africa that have identified a positive correlation between health insurance demand and a household head’s educational or asset-based socioeconomic status [10, 17, 25–27].

Viewed through the prism of ROSCA membership, our results inferred that sociocultural disenfranchisement due to female household headship reduced NHIF demand amongst those not involved in local ROSCAs. However, further analysis revealed that these sociocultural constraints on NHIF enrolment were limited amongst ROSCA members. With this in mind, it is necessary to examine how ROSCA membership changes the terms under which households engage with national power structures.

According to Mladovsky and Mossialos’ social capital framework, bridging capital is vital in building norms and rules to facilitate productive behaviour in individuals [30]. Amongst these norms is the sharing of information and resources amongst socially heterogeneous groups, in order to create a fair market. In the context of ROSCAs, regular social and economic interaction minimizes the information and power asymmetry witnessed in the general population. ROSCAs thus challenge the power structures that are inherent within our study area by developing a common identity under which heterogeneous groups interact. This implies that ROSCAs may foster social cohesion amongst its members, ostensibly by arming different sociocultural groups with the information and financial resources to better understand and afford NHIF membership.

Based on our findings, however, it is, notable from our findings that ROSCA membership does not exclusively negate the influence of sociocultural biases on national health insurance demand. Cognisant of this, we postulate that the social alliances within ROSCAs are ultimately subservient to the economic power held by individuals. It is generally accepted that many ROSCAs exclude those unable to pay their dues during each payment cycle [13]. This means that the wealthy either self-select into the ROSCAs, or ultimately exclude their poorer counterparts due to non-payment of fees [2]. This leads to the pervasive exclusion of the economically marginalised, thus disempowering actors with weak economic agency. The role of potential ostracism in limiting the bridging social capital in ROSCAs is observed by the fact that the wealthier business owners in our study setting, either due to high household head income or multiple earners, were over-represented in ROSCA households. It is for this reason that we found that small-scale business owners were more likely to be enrolled into the NHIF if they had ROSCA membership.

It is important to view the findings of this manuscript within the context of the NHIF’s broader Universal Health Coverage (UHC) strategy in Kenya. Since 2015, the Kenyan Government has provided fully subsidised NHIF coverage to indigents across the country through the Health Insurance Subsidy Programme for the Poor (HISP). This scheme has set the ambitious goal of expanding health insurance coverage to 9 million indigents across the country by 2020 [28]. Additionally, in 2016, the Kenyan Government began offering free maternity services (FMS) to all Kenyan women through the NHIF (Kenya National [23]). With these programmes in mind, the NHIF’s ROSCA targeting strategy would ostensibly be targeted towards a narrow group of informal sector workers who have some degree of economic agency. With this nuanced lens, the NHIF’s strategy of targeting the informal sector through ROSCAs may be feasible, given their alternative means of targeting those at the bottom of the pyramid through premium subsidisation. However, the success of these strategies will be highly dependent upon the successful planning and execution of these collaborative strategies as collective, as well as effectively identifying households at the bottom of the pyramid.

Given the indefinite nature of social capital, care must be taken in viewing the associations reported in this study as causal. We must also acknowledge that the structures of ROSCAs and their relationships with stocks of social capital will vary considerably. We note that ROSCA membership may be influenced by several non-observable factors, such as time-availability and self-selection, which directly impact one’s ability to participate in ROSCAs. We further acknowledge that the use of univariate logistic regression in our study limits our ability to control for some key confounders within our analysis. We therefore reiterate that the relationship between ROSCA membership and its associated social capital, NHIF enrolment and the explanatory variables is associative rather than causative within the context of our study. Further, the urban setting of this study limits its external validity in rural areas. Nevertheless, this article provides an insight into the transmutability of social capital from the local level to national level. It also provides an insight into how an in-depth understanding of the social and economic characteristics of target populations can feed into in the design of national-level schemes. Our study has several design limitations that may affect the external validity of our study: the study took place in the transitory period during which changes to NHIF rates and benefits for the informal sector were being negotiated. Due to this context, the study’s applicability may be affected by the new NHIF voluntary health insurance regime.

## Conclusion

This study examined the influence of ROSCA membership and social capital on voluntary health insurance enrolment. We posit that socially disadvantaged groups may accrue strong bridging social capital by participating in ROSCAs in resource-poor areas. However, we suggest that the full benefits of social capital in increasing health insurance demand can only be accrued by taking into account the underlying economic situation of the target population. We note that the educational and financial situation of a household serves as a strong indicator of its capacity to make investment decisions, including signing up to a voluntary health insurance scheme. It is therefore important to anchor outreach to ROSCAs within a broader, multi-pronged approach that targets households within their social, economic and political realities. These findings are particularly relevant to countries that may seek to increase voluntary health insurance coverage through local associations: it is important to understand the demographic challenges of these groups and utilise several strategies and targeting mechanisms to ensure equitable access to health insurance for all citizens.

## Abbreviations

DHS: Demographic and Health Survey
FMS: Free Maternity Services
HISP: Health Insurance Subsidy Programme for the Poor
NHIF: National Hospital Insurance Fund
NSSF: National Social Security Fund
ODK: Open Data Kit
ROSCAs: Rotating Savings and Credit Associations
SES: Socioeconomic Status
UHC: Universal Health Coverage
